# Integration of traditional and complementary medicine into primary health care systems: a systematic review

**DOI:** 10.2471/BLT.25.293465

**Published:** 2025-09-03

**Authors:** Minmin Wang, Zuokun Liu, Yinuo Sun, Yuyang Zhang, Abdul Ghaffar, Minghui Ren

**Affiliations:** a China Center for Health Development Studies, Peking University, Beijing, China.; b Department of Global Health, School of Public Health, Peking University, No.38 Xueyuan Road, Haidian District, Beijing, 100191, China.; c School of Health Humanities, Peking University, Beijing, China.; d Department of Community Health Sciences, Aga Khan University, Karachi, Pakistan.

## Abstract

**Objective:**

To explore the integration of traditional and complementary medicine in health systems and identify the enablers and barriers to the process.

**Methods:**

We searched PubMed®, Embase, Web of Science, Latin American and Caribbean Health Sciences Literature, China National Knowledge Infrastructure and WanFang Database for original research on integration of traditional and complementary medicine in health systems published from 1 January 2001 to 27 January 2025. We focused on low- and middle-income countries. We made a thematic analysis to identify the enablers of and barriers to integration. We mapped factors according to the six blocks of health-care systems: service delivery; health governance and financing; medical products; health information systems; health workforce; and service standards.

**Findings:**

We included 43 publications from 19 countries, with 55.8% (24/43) from countries in the African Region. Traditional and complementary medicine had the potential to strengthen various aspects of health systems, particularly in health-service delivery and products. We identified 11 determinant domains which could act as both an enabler of and barrier to integration. The most commonly mentioned determinants influencing integration of traditional and complementary medicine were policies and finance, resource availability, and efficacy, quality and safety.

**Conclusion:**

Our findings highlight the role of policies and finance in supporting integration of traditional and complementary medicine, and the need to ensure the quality and safety of traditional products through scientific methods. Reforms in medical education and strategic resource allocation are needed to create the necessary conditions for successful integration of traditional and complementary medicine.

## Résumé

**Objectif:**

Explorer l’intégration de la médecine traditionnelle et complémentaire dans les systèmes de santé et identifier les facteurs favorables et les obstacles à ce processus.

**Méthodes:**

Nous avons effectué des recherches sur PubMed^®^, Embase, Web of Science, Latin American and Caribbean Health Sciences Literature, l’Infrastructure de connaissances nationales de Chine et la base de données WanFang afin de trouver des études originales sur l’intégration de la médecine traditionnelle et complémentaire dans les systèmes de santé publiées entre le 1^er^ janvier 2001 et le 27 janvier 2025. Nous nous sommes concentrés sur les pays à revenu faible et intermédiaire. Nous avons procédé à une analyse thématique afin d’identifier les facteurs favorables et les obstacles à l’intégration. Nous avons cartographié les facteurs en fonction des six éléments des systèmes de santé: prestation de services; gouvernance et financement de la santé; produits médicaux; systèmes d’information sur la santé; personnel de santé; et normes de service.

**Résultats:**

Nous avons inclus 43 publications issues de 19 pays, dont 55,8% (24/43) appartenant à la région d’Afrique. La médecine traditionnelle et complémentaire avait le potentiel de renforcer plusieurs aspects des systèmes de santé, en particulier dans la prestation des services de santé et les produits. Nous avons identifié 11 domaines décisifs qui pouvaient à la fois favoriser et entraver l’intégration de ce type de médecine. Les facteurs décisifs les plus fréquemment mentionnés influençant l’intégration de la médecine traditionnelle et complémentaire étaient les politiques et le financement; la disponibilité des ressources; et l’efficacité, la qualité et la sécurité.

**Conclusion:**

Nos résultats soulignent le rôle des politiques et du financement dans le soutien à l’intégration de la médecine traditionnelle et complémentaire, ainsi que la nécessité de garantir la qualité et la sécurité des produits traditionnels par des méthodes scientifiques. Des réformes de l’enseignement médical et une allocation stratégique des ressources sont nécessaires pour créer les conditions nécessaires à une intégration réussie de la médecine traditionnelle et complémentaire.

## Resumen

**Objetivo:**

Explorar la integración de la medicina tradicional y complementaria en los sistemas de salud e identificar los facilitadores y obstáculos de este proceso.

**Métodos:**

Se realizaron búsquedas en PubMed®, Embase, Web of Science, Literatura Latinoamericana y del Caribe en Ciencias de la Salud, China National Knowledge Infrastructure y la base de datos WanFang de investigaciones originales sobre la integración de la medicina tradicional y complementaria en los sistemas de salud, publicadas entre el 1 de enero de 2001 y el 27 de enero de 2025. El análisis se centró en los países de ingresos bajos y medios. Se efectuó un análisis temático para identificar los facilitadores y obstáculos de la integración. Los factores se mapearon de acuerdo con los seis elementos de los sistemas de salud: prestación de servicios; gobernanza y financiación de la salud; productos médicos; sistemas de información sanitaria; personal de salud; y normas de servicio.

**Resultados:**

Se incluyeron 43 publicaciones de 19 países, de las cuales el 55,8% (24/43) correspondía a países de la Región de África. La medicina tradicional y complementaria mostró potencial para fortalecer diversos aspectos de los sistemas de salud, en particular en la prestación de servicios de salud y los productos médicos. Se identificaron 11 dominios determinantes que podían actuar tanto como facilitadores como obstáculos para la integración. Los determinantes más mencionados que influyeron en la integración de la medicina tradicional y complementaria fueron las políticas y la financiación, la disponibilidad de recursos, y la eficacia, la calidad y la seguridad.

**Conclusión:**

Los hallazgos destacan el papel de las políticas y la financiación en el apoyo a la integración de la medicina tradicional y complementaria, así como la necesidad de garantizar la calidad y seguridad de los productos tradicionales mediante métodos científicos. Son necesarias reformas en la educación médica y una asignación estratégica de recursos para crear las condiciones necesarias que permitan una integración satisfactoria de la medicina tradicional y complementaria.

## ملخص

**الغرض:**

استكشاف دمج الطب التقليدي والتكميلي في الأنظمة الصحية، وتحديد العوامل المساعدة والعقبات التي تواجه هذه العملية.

**الطريقة:**

قمنا بالبحث في PubMed®، وEmbase، وWeb of Science، وأدبيات العلوم الصحية في أمريكا اللاتينية ومنطقة البحر الكاريبي، والبنية التحتية الوطنية للمعرفة في الصين، وقاعدة بيانات WanFang، عن أبحاث أصلية حول دمج الطب التقليدي والتكميلي في الأنظمة الصحية، التي نُشرت في الفترة من 1 يناير/كانون ثاني 2001 إلى 27 يناير/كانون ثاني 2025. قمنا بالتركيز على الدول منخفضة الدخل والدول متوسطة الدخل. قمنا بإجراء تحليل موضوعي لتحديد العوامل المساعدة والعقبات التي تواجه عملية الدمج. وحددنا العوامل وفقًا للعناصر الستة لأنظمة الرعاية الصحية، وهي: تقديم الخدمات؛ وحوكمة وتمويل الصحة؛ والمنتجات الطبية؛ وأنظمة المعلومات الصحية؛ والقوى العاملة الصحية؛ ومعايير الخدمة.

**النتائج:**

قمنا بتضمين 43 منشورًا من 19 دولة، و%55.8 منها (24 من أصل 43) من الدول في المنطقة الأفريقية. كان للطب التقليدي والتكميلي القدرة على تعزيز جوانب مختلفة من النظم الصحية، وبخاصة في تقديم الخدمات الصحية ومنتجاتها. قمنا بتحديد 11 مجالاً معينًا يمكنها أن تكون عاملاً مساعدًا أو معوقًا لعملية الدمج. وكانت أكثر العوامل المُحددة ذكرًا، والتي تؤثر على دمج الطب التقليدي والتكميلي، هي السياسات والتمويل، وتوافر الموارد، والفعالية، والجودة، والسلامة.

**الاستنتاج:**

تُبرز النتائج التي توصلنا إليها دور السياسات والتمويل في دعم دمج الطب التقليدي والتكميلي، والحاجة إلى ضمان جودة وسلامة المنتجات التقليدية من خلال الأساليب العلمية. وهناك حاجة لإجراء إصلاحات في التعليم الطبي، وتخصيص الموارد الاستراتيجية، وذلك بهدف تهيئة الظروف اللازمة لتحقيق دمج ناجح للطب التقليدي والتكميلي.

## 摘要

**目的:**

旨在探讨将传统和补充医学整合到卫生系统这一过程并确认影响这一整合过程的推动因素和障碍。

**方法:**

我们搜索了 PubMed®、Embase、Web of Science、拉丁美洲和加勒比地区卫生科学文献、中国知网以及万方数据库，以查找 2001 年 1 月 1 日至 2025 年 1 月 27 日期间发表的关于将传统和补充医学整合到卫生系统的原始研究数据。我们重点研究了中低收入国家。我们开展了专题分析以确认影响这一整合过程的推动因素和障碍。我们根据卫生保健系统的以下六大要素对各影响因素进行了归类：服务提供、卫生治理和筹资、医疗产品、卫生信息系统、卫生人力和服务标准。

**结果:**

我们纳入了 19 个国家的 43 篇出版物，其中 55.8% (24/43) 的出版物来自非洲区域的国家。传统和补充医学可能有助于加强卫生系统的各个方面，特别是在卫生服务提供和产品方面。我们确定了 11 个决定因素域，这些决定因素域可能会成为这一整合过程的推动因素，也有可能会成为其障碍。最常提及的影响传统和补充医学整合过程的决定因素包括政策和财务、资源可得性以及疗效、质量和安全性。

**结论:**

我们的研究结果强调了政策和财务在支持传统和补充医学整合方面发挥的作用以及通过科学方法确保传统产品质量和安全性的必要性。必须在医学教育和战略资源分配方面进行改革，从而为传统和补充医学的成功整合创造必要条件。

## Резюме

**Цель:**

Изучить вопрос интеграции традиционной и комплементарной медицины в системы здравоохранения и выявить как содействующие, так и препятствующие этому процессу факторы.

**Методы:**

Авторы провели поиск по базам данных PubMed®, Embase, Web of Science, по базе медицинской научной литературы для Латинской Америки и стран Карибского бассейна, а также предприняли поиск в Национальной инфраструктуре знаний Китая и базе данных WanFang, ориентируясь на оригинальные исследования по вопросу интеграции традиционной и комплементарной медицины в системы здравоохранения, опубликованные в период с 1 января 2001 года по 27 января 2025 года. Основное внимание уделялось странам с низким и средним уровнем доходов. Для выявления факторов, способствующих и препятствующих такой интеграции, был проведен тематический анализ. Эти факторы были отнесены к шести элементам системы здравоохранения: оказание услуг, управление здравоохранением и его финансирование, медицинская продукция, системы информации в здравоохранении, медицинский персонал и стандарты оказания услуг.

**Результаты:**

В обзор вошли 43 публикации из 19 стран, из которых 55,8% (24/43) приходилось на страны Африканского региона. Традиционная и комплементарная медицина обладает потенциалом для укрепления различных аспектов систем здравоохранения, в частности в том, что касается оказания услуг и медицинской продукции. Авторы выявили 11 областей-детерминант, которые могут проявляться одновременно как содействующие и препятствующие факторы на пути внедрения. Наиболее часто упоминаемыми детерминантами, которые влияют на интеграцию традиционной и комплементарной медицины, были вопросы политики и финансирования, доступность ресурсов, а также эффективность, качество и безопасность.

**Вывод:**

Результаты подчеркивают роль политических и финансовых факторов в поддержке интеграции традиционной и комплементарной медицины, а также указывают на потребность в гарантированном качестве и безопасности традиционной продукции, которые должны достигаться научными методами. Для создания необходимых условий успешной интеграции традиционной и комплементарной медицины необходимы реформы в медицинском образовании и выделение стратегических ресурсов.

## Introduction

Traditional and complementary medicine includes a wide range of health practices and products rooted in the cultural beliefs, theories and experiences of various populations.^1^ These practices, whether scientifically explainable through the research gold standard (randomized controlled trials) or not, are used to maintain health, and prevent, diagnose and treat both physical and mental illnesses in almost all countries of the world. Traditional and complementary medicine is deeply embedded in traditional knowledge, cultural practices, histories and geographical contexts, especially in low- and middle-income countries.^2^^–^^4^ Such medicine is a valuable yet often underappreciated health-care resource, particularly in the prevention and management of lifestyle-related chronic diseases and in addressing the health needs of ageing populations.

The integration of traditional and complementary medicine into health systems, particularly in primary health care, is increasingly seen as a key strategy for advancing universal health coverage (UHC) and addressing global health challenges, with the aim of achieving health equity worldwide.^5^^,^^6^ By 2018, 98 Member States of the World Health Organization (WHO) had developed national policies on traditional and complementary medicine, 109 had enacted national laws or regulations, and 124 had introduced regulations governing herbal medicines.^7^ The *WHO traditional medicine strategy 2014–2023*^8^ was introduced in response to World Health Assembly resolution WHA62.13, with the objective of supporting Member States in the development of policies and action plans to enhance the role of traditional and complementary medicine in public health. Another key objective of this global strategy was to promote UHC by integrating traditional and complementary medicine into health-care services and self-care practices. Similarly, the *Regional framework for harnessing traditional and complementary medicine for achieving health and well-being in the Western Pacific*^9^ was developed to highlight traditional and complementary medicine’s contribution to tackling noncommunicable diseases, ageing populations and disparities in health-care access.

Despite these promising developments, progress in integration of traditional and complementary medicine into primary health care is slow, mainly because of the lack of clarity on the facilitators and barriers to this process, especially in low- and middle-income countries. This problem impedes the identification of effective pathways for integration of traditional and complementary medicine. For instance, the *WHO global report on traditional and complementary medicine 2019*^10^ indicated that seminars and workshops focused on this integration were a priority for many Member States. However, evidence on integration was limited and focused on only a few countries or regions.^11^^,^^12^ The situation has not improved because research is lacking, mainly due to methodological and funding challenges.^2^ To motivate the process, in 2025, the WHO Executive Board mandated the WHO Secretariat to develop guidance on the integration of safe and effective traditional and complementary medicine practices into national health systems. The Executive Board called for the establishment of standardized indicators to monitor access to, coverage and use of traditional and complementary medicine practices, and assessment of their safety and effectiveness, based on WHO’s traditional medicine strategy for 2025–2034.^13^

To address the research gaps, we conducted a systematic review to: (i) explore how integration of traditional and complementary medicine can strengthen health systems; and (ii) identify the factors that influence this integration in low- and middle-income countries. The findings of this review will provide scientific and policy insights to facilitate the integration of traditional and complementary medicine into health-care systems and thereby help advance the achievement of UHC and health equity in low- and middle-income countries.

## Methods

We conducted this review using the Preferred Reporting Items for Systematic Review and Meta-Analyses guidelines. We registered the review with PROSPERO (CRD420250654426).

### Search strategy

We searched PubMed®, Embase, Web of Science, Latin American and Caribbean Health Sciences Literature, China National Knowledge Infrastructure and WanFang Database. The search period was 1 January 2001 to 27 January 2025. We used Medical Subject Headings and free-text identifiers. Search terms covered three main areas: traditional and complementary medicine; health systems; and low- and middle-income countries. We used the World Bank country classifications by income level for 2024–2025 to classify low- and middle-income countries.^14^ The complete search strategy is given in the online repository.^15^

### Eligibility criteria

Inclusion criteria were: (i) original research articles; (ii) studies conducted in low- and middle-income countries; (iii) publications from 2001 onwards; and (iv) studies with an explicit focus on factors affecting the integration of traditional and complementary medicine into the health-care system. We focused on papers published from 2001 onwards because WHO published guidelines then advocating the integration of traditional medicine into modern health-care systems and began adopting the term traditional and complementary medicine.^16^

Four authors independently screened titles and abstracts. Subsequently, two reviewers conducted full-text assessments to select eligible articles. We did not apply any language restrictions. We used a translation tool (DeepL SE, Cologne, Germany) to translate articles that were published in languages other than English or Chinese. Two authors independently assessed the methodological quality and risk of bias of the studies included by applying the recommendations of the United States Agency for Healthcare Research and Quality and determining a related score.^17^ We calculated the score from 11 quality indicators: a score of 0–4, 5–7 or 8–11 indicates a high, moderate or low risk of bias, respectively.

### Data extraction and synthesis

#### Thematic analysis

We used a thematic analysis to collect and evaluate extracted data.^18^ We applied a standardized framework to extract data on factors affecting the process and outcome of integration, which we categorized as facilitators or barriers. We assigned factors as facilitators and barriers based on whether the author or participant in the original study considered the factor to play a positive or negative role in integration. We adapted an established framework (the conceptual framework for integration of traditional medicine with national health-care systems)^5^ to identify information that needed to be extracted. We extracted four main dimensions from the framework, including: (i) historical and cultural use of traditional and complementary medicine; (ii) resource availability; (iii) attitude to and acceptance by (traditional or regular health-care service) providers; and (iv) policies and finance. We anticipated that additional dimensions would emerge during full-text analysis and data extraction, so we prospectively mapped potential themes using simple descriptors, for example, education, quality, safety and competition. We also systematically mapped extracted factors according to the six blocks of health-care systems: service delivery; health governance and financing; medical products; health information systems; health workforce; and service standards. Two authors independently extracted the information, with disagreements resolved by consensus.

#### Data analysis

We did a descriptive analysis of the articles extracted, including of their basic characteristics (e.g. time and place) and distribution of articles across socioeconomic dimensions and health system blocks. We undertook a thematic analysis of the influencing factors to characterize their roles (as enablers or barriers), distribution across dimensions and alignment with health system blocks. The different factors were explored in relation to strengthened health system blocks. We developed a conceptual framework to elucidate which sociostructural dimensions, through which health system blocks and under what contextual conditions, collectively influence the integration of traditional and complementary medicine into national health-care systems.

## Results

### Study selection 

**Fig. 1:**
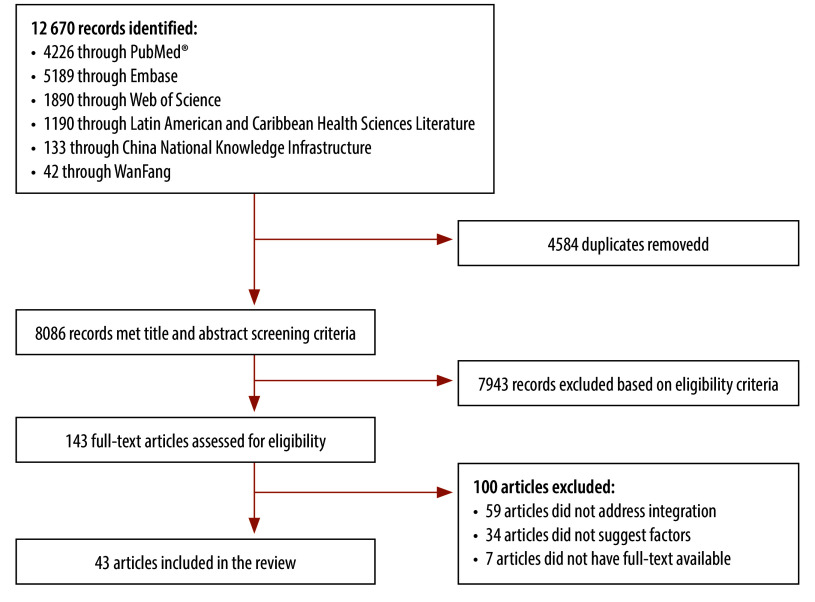
Flowchart of selection of papers on integration of traditional and complementary medicine

The initial search yielded 12 670 records. After removal of duplicates, we screened titles and abstracts of 8086 records, with 143 advancing to full-text review. In the end, 43 studies met the eligibility criteria (Fig. 1).^19^^–^^61^

Basic information of the 43 studies are summarized in Table 1; available at https://www.who.int/publications/journals/bulletin/. About half of the articles (20; 46.5%) were published between 2021 and 2025. Geographically, 42 studies came from 19 countries: the Plurinational State of Bolivia,^19^ Brazil,^20^ China,^21^^–^^23^ Democratic Republic of the Congo,^24^ Eritrea,^25^ Ethiopia,^26^^,^^27^ Georgia,^28^ Ghana,^29^^–^^37^ India,^38^^–^^44^ Indonesia,^45^ Kenya,^46^ Malaysia,^47^ Nigeria,^48^ Papua New Guinea,^49^ South Africa,^50^^–^^54^ Thailand,^55^^,^^56^ Uganda,^57^^,^^58^ Vanuatu^59^ and Zimbabwe.^60^ The remaining article examined three African countries, Ghana, Kenya and Nigeria.^61^ Of the 43 articles, 24 (55.8%) were from countries in the WHO African Region, so we did a subgroup analysis on this set. Overall, 14 studies (32.6%) focused on upper-middle income countries, 23 (53.5%) on lower-middle income countries and six (14.0%) on low-income countries.

**Table 1:** Characteristics of the studies included in the review of integration of traditional and complementary medicine into health systems

Author, year, by country	Study design	Study population	Disease	Disease staging	Quality score^a^	Risk of bias
**Bolivia, (Plurinational State of)**						
Torri, 2013^19^	Qualitative	Health providers, policy-makers, service users	General	General	7	Moderate
**Brazil**						
Ischkanian, 2012^20^	Qualitative	Health providers, policy-makers	General	General	6	Moderate
**China**						
Meng, 2022^21^	Quantitative	Service users	Chronic noncommunicable diseases	Prevention, treatment	8	Low
Fang, 2023^22^	Quantitative	Health providers, policy-makers	General	General	7	Moderate
Zhou, 2024^23^	Quantitative	NA	General	General	NA	NA
**Democratic Republic of the Congo**						
Mutombo, 2022^24^	Qualitative	Service users	General	Treatment	8	Low
**Eritrea**						
Habtom, 2015^25^	Mixed methods	Health providers	General	General	7	Moderate
**Ethiopia**						
Legesse, 2023^26^	Qualitative	Service users	General	General	6	Moderate
Mohammed, 2024^27^	Quantitative	Service users	Maternal health	Prevention, treatment	8	Low
**Georgia**						
Nadareishvili, 2019^28^	Qualitative	Health providers	General	General	10	Low
**Ghana**						
Agyei-Baffour, 2017^29^	Quantitative	Service users	General	General	6	Moderate
Ampomah, 2025^30^	Qualitative	Health providers	Malaria	Treatment	7	Moderate
Ampomah, 2021^31^	Qualitative	Health providers	General	General	8	Low
Ampomah, 2022^32^	Mixed methods	Service users	General	General	8	Low
Ampomah, 2023^33^	Qualitative	Health providers	General	General	8	Low
Boateng, 2016^34^	Qualitative	Health providers, policy-makers	General	General	5	Moderate
Gyasi, 2017^35^	Qualitative	Health providers, service users	General	General	6	Moderate
Krah, 2018^36^	Qualitative	Health providers, policy-makers, service users	General	General	4	High
Kwame, 2021^37^	Qualitative	Health providers, policy-makers, service users	General	General	7	Moderate
**Ghana, Kenya, Nigeria**						
van der Watt, 2017^61^	Qualitative	Health providers	Mental health	General	8	Low
**India**						
Nambiar, 2014^38^	Qualitative	Health providers, policy-makers and service users	General	General	5	Moderate
Nisula, 2006^39^	Mixed methods	Health providers	General	Treatment, management	2	High
Patel, 2023^40^	Qualitative	Health providers, policy-makers	General	General	7	Moderate
Bhargav, 2022^41^	Qualitative	Policy-makers	General	General	2	High
Singhal, 2018^42^	Qualitative	Health providers	General	General	10	Low
Dehury, 2016^43^	Qualitative	Health providers, service users	Maternal health	General	6	Moderate
Patel, 2021^44^	Qualitative	Health providers, policy-makers	General	General	7	Moderate
**Indonesia**						
Liem, 2020^45^	Qualitative	Health providers	Mental health disorders	Prevention, treatment, management	6	Moderate
**Kenya**						
Ong’udi, 2019^46^	Qualitative	Service users	Cancer	Treatment, management	6	Moderate
**Malaysia**						
Park, 2022^47^	Qualitative	NA	General	Prevention, treatment, management	NA	NA
**Nigeria**						
Awodele, 2011^48^	Quantitative	Health providers	General	General	5	Moderate
**Papua New Guinea**						
Macfarlane, 2010^49^	Quantitative	Health providers	General	Prevention, treatment, management, diagnosis	6	Moderate
**South Africa**						
Lawrence, 2021^50^	Qualitative	Health providers	General	General	5	Moderate
Pinkoane, 2012^51^	Qualitative	Service users	General	Prevention, treatment, management	5	Moderate
Peu, 2001^52^	Mixed methods	Health providers	General	Prevention	6	Moderate
Masemola, 2023^53^	Quantitative	Health providers	Mental health	General	6	Moderate
Mutola, 2021^54^	Qualitative	Health providers, service users	General	General	6	Moderate
**Thailand**						
Nootim, 2019^55^	Qualitative	Service users	Cancer (specifically liver cancer in stages III and IV)	Treatment	8	Low
Suwankhong, 2011^56^	Qualitative	Health providers, policy-makers, service users	General	General	7	Moderate
**Uganda**						
Kyeyune, 2024^57^	Qualitative	Health providers	General	Diagnosis, screening, treatment	7	Moderate
Mwaka, 2015^58^	Qualitative	Service users	Cervical cancer	Prevention, management	8	Low
**Vanuatu**						
Viney, 2014^59^	Mixed methods	Health providers	Tuberculosis	Treatment	8	Low
**Zimbabwe**						
Mudonhi, 2021^60^	Qualitative	Health providers	Maternal health	Prevention, screening	5	Moderate

NA: not applicable.

^a^ We used the recommendation from the United States Agency for Healthcare Research and Quality to assess the methodological quality of the studies.^17^ A score of 0–4 indicates a high risk of bias, 5–7 a moderate risk and 8–11 a low risk.

Regarding the types of diseases or conditions studied, 32 (74.4%) studies broadly addressed general diseases which were treatable by traditional and complementary medicine. Overall, 11 of the 43 articles targeted specific diseases: cancer (three; 7.0%);^46^^,^^55^^,^^58^ mental disorders (three; 7.0%);^45^^,^^53^^,^^61^ malaria (one; 2.3%);^30^ tuberculosis (one; 2.3%);^59^ and maternal health (three; 7.0%).^27^^,^^43^^,^^60^ Most studies (28; 65.1%)^20^^,^^22^^,^^25^^–^^26^^,^^28^^,^^30^^–^^33^^,^^35^^–^^38^^,^^46^^–^^54^^,^^56^^–^^61^ used the term traditional and complementary medicine, or its subcategories and derivations, such as alternative medicine or traditional healers. Overall, 20.1% (9/43)^21^^,^^24^^,^^39^^–^^44^^,^^55^ of the articles studied specific traditional and complementary medical practices rooted in a particular culture, such as traditional Chinese medicine or ayurveda; six (14.0%)^19^^,^^23^^,^^27^^,^^29^^,^^34^^,^^45^ articles used concepts or techniques with intersection with complementary medicine, such as integrative medicine or herbal medicine. Most articles (30; 69.8%)^19^^,^^20^^,^^24^^,^^26^^,^^28^^,^^30^^,^^31^^,^^33^^–^^38^^,^^40^^–^^47^^,^^50^^,^^51^^,^^54^^–^^58^^,^^60^^,^^61^ reported qualitative research, with eight (18.6%)^21^^–^^23^^,^^27^^,^^29^^,^^48^^,^^49^^,^^53^ reporting quantitative research and five (11.6%)^25^^,^^32^^,^^39^^,^^52^^,^^59^ using mixed methods. Finally, 31 (72.1%)^19^^,^^20^^,^^22^^,^^25^^,^^28^^,^^30^^,^^31^^,^^33^^–^^40^^,^^42^^–^^44^^,^^47^^–^^50^^,^^52^^–^^54^^,^^56^^,^^57^^,^^59^^–^^61^ articles included health providers as research participants; 18 (41.9%)^19^^,^^21^^,^^24^^,^^26^^,^^27^^,^^29^^,^^32^^,^^35^^–^^38^^,^^43^^,^^46^^,^^51^^,^^54^^–^^56^^,^^58^ articles included service users; and 11 (25.6%)^19^^,^^20^^,^^22^^,^^34^^,^^36^^–^^38^^,^^40^^,^^41^^,^^44^^,^^56^ included policy-makers.

### Role in strengthening health systems

**Fig. 2:**
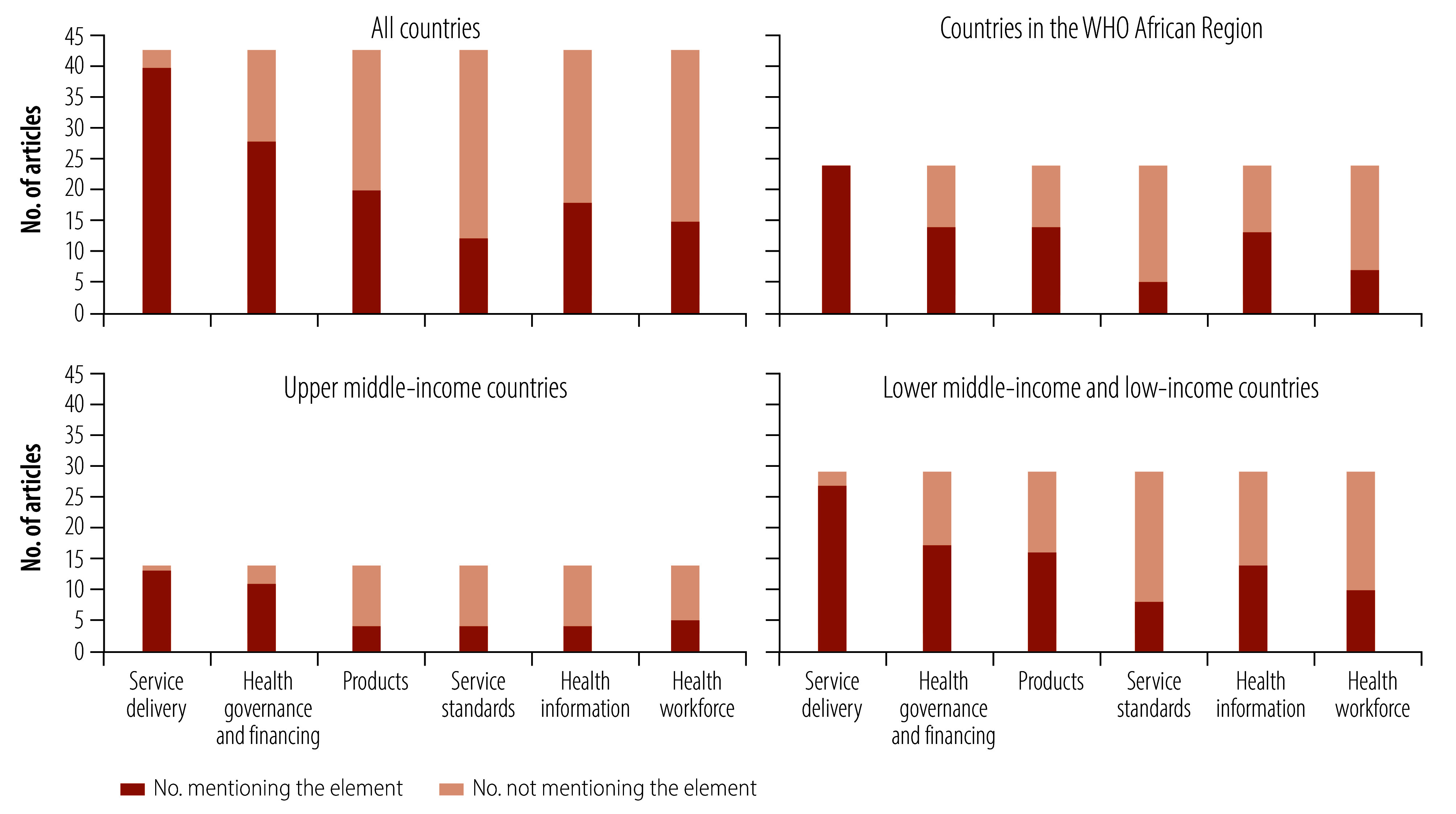
Health system blocks mentioned in 43 articles on integration of traditional and complementary medicine

We assessed the number of articles that referred to the role of traditional and complementary medicine in strengthening the six blocks of health systems (Fig. 2). Among all studies, 93.0% (40/43)^19^^–^^32^^,^^34^^–^^43^^,^^45^^–^^60^ referred to service delivery, which was the most cited element; followed by 65.1% (28/43)^19^^–^^27^^,^^29^^,^^31^^–^^35^^,^^37^^,^^39^^–^^41^^,^^43^^,^^44^^,^^49^^,^^51^^,^^54^^,^^56^^,^^57^^,^^59^^,^^61^ related to health governance and financing; and 46.5% (20/43)^19^^,^^23^^–^^29^^,^^33^^–^^35^^,^^37^^–^^40^^,^^42^^,^^49^^,^^51^^,^^52^^,^^57^ related to medical products. Among the studies in the African Region, 58.3% (14/24)^24^^–^^27^^,^^29^^,^^33^^–^^35^^,^^37^^,^^51^^–^^53^^,^^57^ referred to medical products, and 54.1% (13/24)^24^^–^^27^^,^^29^^–^^32^^,^^48^^,^^52^^,^^57^^,^^58^^,^^60^ to health information systems. 

WHO: World Health Organization.

In upper-middle income countries, 78.6% (11/14)^20^^–^^23^^,^^32^^,^^43^^,^^45^^,^^47^^,^^51^^,^^54^^,^^56^ of articles referred to health governance and financing, while 28.6% (4/14)^23^^,^^28^^,^^51^^,^^52^ each referred to medical products, health information or service standards. In lower-income countries, 55.2% (16/29)^19^^,^^24^^–^^27^^,^^33^^-^^35^^,^^37^^–^^40^^,^^42^^,^^49^^,^^51^^,^^57^ of articles referred to medical products and 48.3% (14/29)^19^^,^^24^^,^^25^^,^^27^^,^^29^^,^^30^^,^^38^^–^^40^^,^^42^^,^^48^^,^^57^^,^^58^^,^^60^ to health information.

### Integrating traditional medicine

#### Determinants 

After thematic synthesis, we identified 11 domains affecting the integration of traditional and complementary medicine into the biomedicine system: (i) attitude and acceptance by providers, that is, the attitude towards and acceptance of traditional and complementary medicines by health workers; (ii) attitude and acceptance by users, that is, the attitude and acceptance of traditional and complementary medicine and the biomedicine system by patients; (iii) communication and cooperation, that is, personnel communication and departmental cooperation between the traditional and biomedical health systems; (iv) competition, that is, the competitive relationship between traditional and biomedical health systems; (v) medical education, that is, higher education in medicine in universities, including education on the biomedical and traditional health systems; (vi) historical and cultural use of traditional and complementary medicine; (vii) policies and finance, that is, whether the policy and finance provide support for traditional medicine; (viii) efficacy, quality and safety, that is, treatment effect, adverse reaction and interactions with other medicines and products; (ix) guidelines and standards, that is, industrial standards, medical staff licences, standard operating procedures and market approval for drugs; (x) resource availability, that is, availability of health services, human resources and medicines; and (xi) others (e.g. publicity, market strategy and privacy protection).

#### Determinant framework

**Fig. 3:**
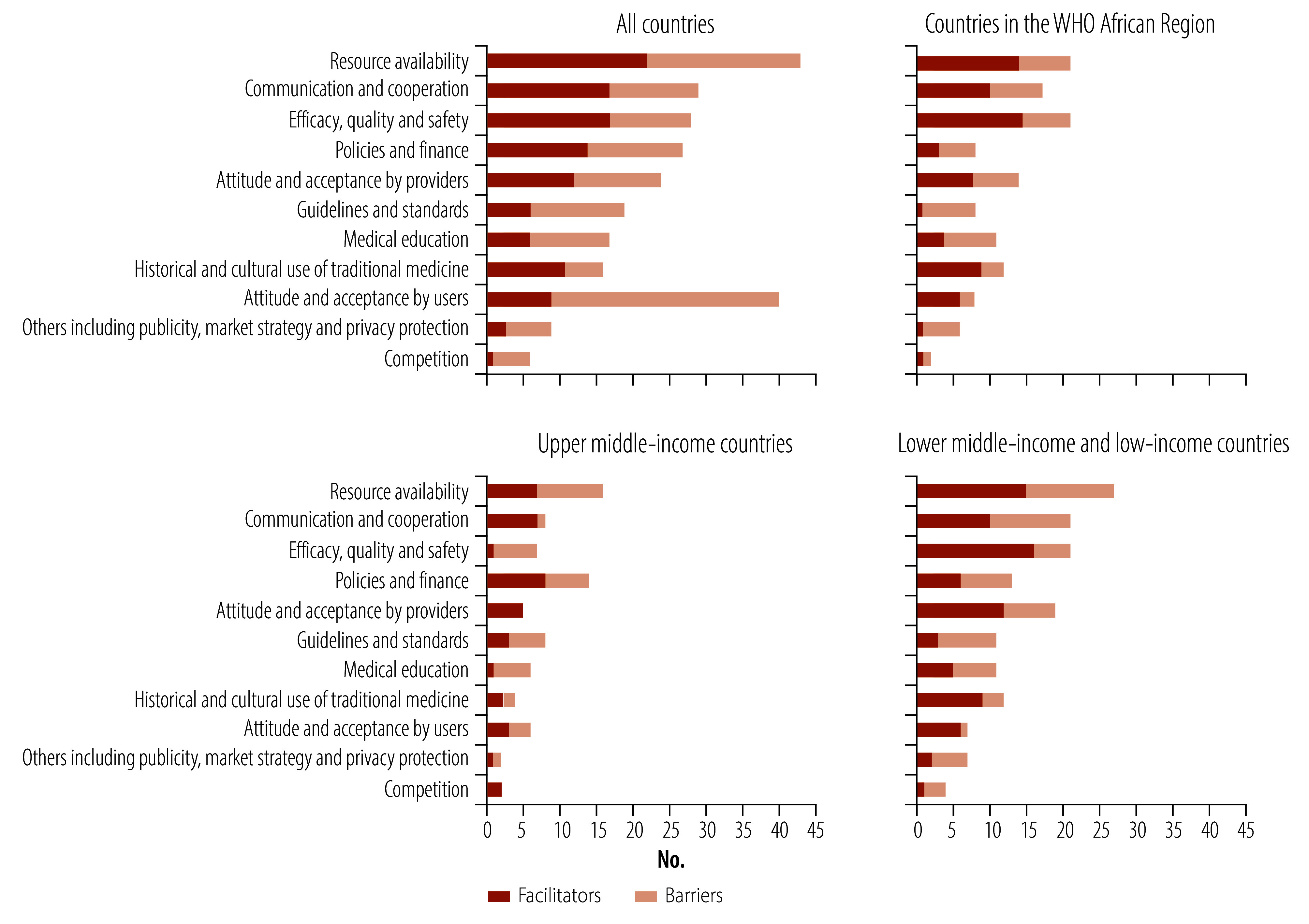
Facilitators of and barriers to integration of traditional and complementary medicine mentioned in articles

We extracted 231 barriers and enablers (Table 2; available online at https://www.who.int/publications/journals/bulletin; and online repository).^15^ In all countries, resource availability was the most frequently cited dimension (18.6%; 43/231), followed by communication and cooperation (12.6%; 29/231) and efficacy, quality and safety (12.1%; 28/231). In contrast, attitude and acceptance by users (5.6%; 13/231), others (3.9%; 9/231) and competition (2.6%; 6/231) were the least-referenced dimensions. Notable deviations emerged across the subgroups (Fig. 3). In studies in the African Region, efficacy, quality and safety (16.4%; 21/128) and resource availability (16.4%; 21/128) were most frequently cited. After resource availability, upper-middle income countries prioritized policies and finance (17.9%; 14/78) compared with lower-middle income and low-income countries (8.5%; 13/153); while lower-middle income and low-income countries more frequently cited efficacy, quality and safety (13.7%; 21/153 versus 9.0%; 7/78), and communication and cooperation (10.3%; 8/78 versus 13.7%; 21/153).

**Table 2:** Identified barriers and enablers of integration of traditional and complementary medicine into health systems

Health system block	No. of studies	
	Barrier	Enabler
**Service standard**		
Guidelines and standards	7^24^^,^^31^^,^^35^^,^^38^^,^^43^^,^^54^^,^^55^^,^^57^	4^41^^,^^45^^,^^47^^,^^49^
**Service delivery**		
Resource availability	7^20^^,^^21^^,^^23^^,^^30^^,^^38^^,^^45^^,^^47^	14^21^^,^^22^^,^^24^^,^^27^^,^^29^^,^^32^^,^^39^^,^^40^^,^^45^^,^^46^^,^^49^^,^^52^^,^^58^^,^^60^
Historical and cultural use of traditional medicine	2^19^^,^^43^	4^24^^,^^36^^,^^38^^,^^57^
Efficacy, quality and safety	2^23^^,^^37^	7^26^^,^^27^^,^^29^^,^^31^^,^^39^^,^^48^^,^^52^
Competition	4^37^^,^^45^^,^^47^^,^^49^	1^60^
Communication and cooperation	5^19^^,^^33^^,^^35^^,^^36^^,^^49^	5^25^^,^^28^^,^^36^^,^^38^^,^^61^
Attitude and acceptance by users	3^28^^,^^51^^,^^58^	9^19^^,^^21^^,^^24^^,^^31^^,^^39^^,^^46^^,^^52^^,^^54^^,^^58^
Attitude of and acceptance by all providers	7^30^^,^^34^^,^^38^^,^^42^^,^^50^^,^^53^^,^^56^	11^25^^,^^30^^,^^32^^,^^35^^,^^37^^,^^39^^,^^40^^,^^48^^,^^49^^,^^59^^,^^60^
**Products**		
Resource availability	4^30^^,^^31^^,^^36^^,^^40^	2^24^^,^^26^
Guidelines and standards	2^33^^,^^47^	1^57^
Efficacy, quality and safety	7^24^^,^^42^^,^^46^^,^^47^^,^^54^^,^^55^^,^^57^	8^24^^,^^27^^,^^32^^,^^49^^,^^51^^,^^57^^,^^60^^,^^61^
Others^a^	NA	1^39^
**Health workforce**		
Resource availability	4^22^^,^^30^^,^^42^^,^^55^	5^31^^,^^39^^,^^41^^,^^48^^,^^50^
Medical education	7^30^^,^^31^^,^^33^^,^^35^^,^^50^^,^^55^^,^^56^	NA
Guidelines and standards	2^55^^,^^56^	NA
Attitude and acceptance by providers	5^36^^,^^47^^,^^49^^,^^55^^,^^61^	NA
**Health information**		
Medical education	2^42^^,^^43^	1^61^
Historical and cultural use of traditional medicine	2^52^^,^^61^	3^46^^,^^49^^,^^58^
Communication and cooperation	1^31^	3^27^^,^^51^^,^^57^
Others^a^	6^20^^,^^26^^,^^32^^–^^34^^,^^60^	2^21^^,^^58^
**Health governance and financing**		
Resource availability	4^31^^,^^33^^,^^40^^,^^55^	NA
Policies and finance	12^20^^,^^21^^,^^23^^,^^30^^,^^34^^,^^35^^,^^41^^,^^47^^,^^49^^,^^56^^,^^60^^,^^61^	11^21^^,^^22^^,^^36^^,^^38^^–^^41^^,^^47^^,^^49^^–^^51^
Historical and cultural use of traditional medicine	1^51^	4^27^^,^^50^^,^^52^^,^^60^
Competition	1^44^	NA
Communication and cooperation	4^38^^,^^45^^,^^46^^,^^61^	6^41^^,^^45^^,^^49^^,^^51^^,^^52^^,^^57^

NA: not applicable.

^a^ For example, publicity, market strategy and privacy protection.

Note: more details about the barriers and enablers are available in the online repository.^15^

WHO: World Health Organization.

**Fig. 4:**
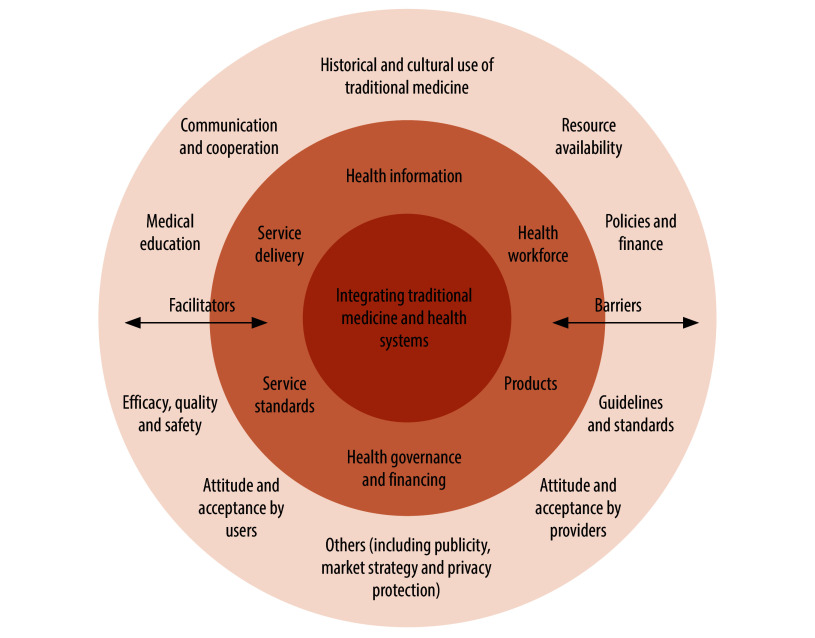
Framework of factors influencing integration of traditional and complementary medicine in health systems

Note: The middle ring shows the six health system blocks. The outer ring shows the 11 sociostructural dimensions influencing integration.

We constructed a concentric circular framework (Fig. 4) to delineate the interplay between sociostructural dimensions and health system blocks in shaping the integration of traditional medicine into health systems. The framework has three layers: the inner circle represents the core objective of integrating traditional and complementary medicine into the health system; the middle ring shows the six modifiable health system blocks through which integration pathways are facilitated; and the outer ring shows the 11 sociostructural dimensions that influence the integration process. The influence of these factors, as either facilitators or barriers, is contingent on contextual interventions, policy formulation and stakeholder collaboration.

#### Key enablers and barriers

Of the 231 factors, 118 (51.1%) were facilitators and 113 (48.9%) barriers. All 11 dimensions had dual roles, acting as either facilitators or barriers depending on contextual variables. The studies in the African Region most noted the absence of guidelines and standards as a systemic barrier to integration of traditional and complementary medicine. In upper-middle income countries, communication and cooperation was clearly characterized as a facilitator, whereas competition and medical education were consistently perceived as barriers. Conversely, lower-middle income and low-income countries uniformly identified attitude and acceptance of users as an important facilitator.

The determinants were further analysed by the role of traditional and complementary medicine in health system strengthening. The 231 factors were systematically cross-mapped to the six blocks of health systems and their associated dimensions to reveal pathways through which sociostructural dimensions influence integration trajectories (online repository).^15^ The medical product element was mainly influenced by efficacy, quality and safety, while health governance and financing was mostly affected by policies and finance. Service delivery was influenced by a wide range of dimensions, including resource availability, and attitude and acceptance by providers.

#### Sensitivity analysis

After conducting quality assessment, we did a sensitivity analysis by removing five documents^23^^,^^36^^,^^39^^,^^41^^,^^47^ with high-risk assessment and inapplicable evaluation methods (online repository).^15^ We obtained robust results about the role of health system blocks, and the facilitators of and barriers to integration.

## Discussion

In this review, we analysed the potential contribution of traditional and complementary medicine to strengthening health systems. Our results could provide evidence-based guidance for countries to accelerate the integration of traditional and complementary medicine into primary health care and for national health systems to achieve the commitment to UHC.

Our results show that policies and finance was widely acknowledged as an important determinant (whether enabler or barrier) of the integration of traditional and complementary medicine into health-care systems, particularly in low- and middle-income countries. First, the global commitment advocates incorporating traditional and complementary medicine into health systems, with the recognition that it plays an important role in advancing the goal of UHC.^10^ For instance, research on the use of traditional and herbal medicines in members of the Association of Southeast Asian Nations^62^ underscored WHO’s pivotal role in advocating for the integration of traditional and complementary medicine into national health-care infrastructures, thereby promoting a more inclusive and holistic approach to health. Second, at the national level, policies and finance often covers concrete policy measures, such as the enactment of legislation, the formulation of supportive policies, financial investments by governments and interorganizational collaborations. An integrative health-care system is inherently complex,^63^ requiring a multifaceted approach that combines both local needs and global priority areas. Thus, the successful integration of traditional and complementary medicine into health-care systems hinges on the alignment of domestic policies with global frameworks and also the sustained political commitment to fostering such systems at both governmental and organizational levels.^47^^,^^64^

Resource availability is one of the most frequently mentioned determinants influencing the integration of traditional and complementary medicine into health-care systems, particularly in low- and middle-income countries. Interestingly, this determinant is often cited as a key enabler of integration, primarily due to the advantages that traditional and complementary medicine offers in resource-constrained settings. For example, the geographic accessibility and financial affordability of traditional and complementary medicine services provide a compelling rationale for their inclusion in primary health-care systems. In China, acupuncture and moxibustion have been incorporated into various health insurance programmes,^65^ reflecting the recognition of these traditional medicines as a cost-effective health-care option. Similarly, studies have shown that the affordability and relatively low cost of traditional and complementary medicine significantly influence patients’ decisions to seek such treatments. Furthermore, the widespread availability of traditional and complementary medicine practitioners, especially in underserved and rural areas, positions it as an important first-contact service within the local health system.^49^ However, despite its potential advantages, resource availability can also present challenges to the integration of traditional and complementary medicine. Key obstacles include the difficulties associated with the lack of precise traditional medical diagnostic tools; lack of standardized training for practitioners of traditional and complementary medicine; weak referral systems; and the inconsistent supply of herbal products and other necessary facilities, such as clean clinics, hospital beds and medical equipment.^31^^,^^66^ These issues were identified as important barriers in several studies, emphasizing the complexity of constructing an integrative health-care system. The successful integration of traditional and complementary medicine into health systems requires balancing the advantages of resource availability with the need for rigorous oversight and standardization to ensure that such practices can contribute safely and effectively to the broader health-care framework.

Efficacy, quality and safety is a key concern with the use of traditional and complementary medicine in health-care systems, especially in regard to medical products used in traditional medicine. The trust, effectiveness and cultural significance of these products, which are often deeply rooted in local traditions, help make them more accepted in primary health care. For example, a study in Ghana found that most people considered practitioners of traditional and complementary medicine more caring and empathetic than regular doctors.^32^ This sense of familiarity and compassion was seen as a reason to include traditional and complementary medicine in broader health-care systems. However, concerns remain about the efficacy, quality and safety of traditional and complementary medicine, including about possible side-effects, lack of scientific proof and the absence of quality standards. These issues have always been barriers to using traditional and complementary medicine more widely.^55^^,^^57^ In addition, in a survey of 133 countries, 99 respondents reported a lack of research data on traditional and complementary medicine, and 75 respondents said that the lack of safety checks was grave concern.^3^ To address these issues, establishing clear guidelines for researching, evaluating and monitoring the safety, quality and effectiveness of traditional and complementary medicine is a priority. Some progress has been made in solving these problems, especially in quality and safety. For example, the WHO South-East Asia Region’s workshop on pharmacovigilance^67^ focused on improving the reporting of side-effects of traditional and complementary medicines. This project aimed to help countries in the region track and report possible side-effects using shared knowledge and best practices; identify the regional and country priority action points; and evaluate technical areas that can provide support to strengthen pharmacovigilance to improve safety monitoring of traditional and complementary medical products.

Further concerns have been raised about the need to reform medical education and the competition between health systems. Medical education plays a key role in the integration of traditional and complementary medicine. It is therefore important to include traditional and complementary medicine in medical school curricula. This inclusion will help trainee doctors understand and collaborate with traditional healers. A lack of a formal curriculum and the informal basis of training in traditional and complementary medicine can cause distrust in traditional practices and hinder integration efforts.^44^ Another issue is the competition between health systems, especially between the medicine system and traditional and complementary medicine. The different approaches to treatment, concerns about the boundaries of the scope of each system, and the lack of effective communication between practitioners create a fragmented health-care environment. Several studies have observed the negative perceptions and attitudes of health workers towards the integration of traditional and complementary medicine.^30^^,^^50^^,^^55^ Some health workers were reportedly resistant to making changes, thus causing conflict between traditional health practitioners and modern health services.^44^ These issues underscore the need for policy coherence and the establishment of collaborative frameworks to harness the complementary strengths of both systems and optimize patient care.

A strength of our study is that it used a new approach to clustering the barriers to and enablers of integration of traditional and complementary medicine into primary health care and health systems in low- and middle-income countries. Our study also has limitations. First, we could not rule out the influence of the selective reporting of positive or negative results. Second, although we searched six databases with no language restrictions, potentially relevant studies catalogued elsewhere were not considered.

In conclusion, our study provides a greater understanding of the role that traditional and complementary medicine can play in primary health care and the broader health system, and of the enablers and facilitators that can promote integration of these systems.
